# A Modified Differential Evolution Algorithm Based on Improving A New Mutation Strategy and Self-Adaptation Crossover

**DOI:** 10.1016/j.mex.2023.102276

**Published:** 2023-06-30

**Authors:** Sadeer Fadhil, Hegazy Zaher, Naglaa Ragaa, Eman Oun

**Affiliations:** a Operations Research, Faculty of Graduate Studies for Statistical Research; b Mathematical Statistics, Faculty of Graduate Studies for Statistical Research

**Keywords:** Optimization, Heuristics, Metaheuristics, Differential evolution algorithm, Design of experiments, Modified Differential Evolution Algorithm for Solving Optimization Problems

## Abstract

The differential evolution algorithm is one of the promising natural inspired population-based metaheuristic algorithms that attracted the attention of researchers in the recent years. This paper presents a new mutation strategy called DE/current-to-best/2 that presents a new mutated vector based on utilizing the distance between the best vector and the current vector along with another random vector. In addition, the crossover procedure is self-adapted to cover low locality and high locality based on the iteration number. To obtain the best results of the proposed modified differential evolution algorithm, design of experiments is done to optimize its parameters. The comparative results are done using 11 optimization problems to compare the classical version of differential evolution algorithm with the new modified version and the results show high efficiency of the proposed DE algorithm in terms of CPU time, evaluation, and accuracy

The outline of the work done in this paper can be shown as follows:

• The paper produces a new modification of one of the most promising metaheuristics algorithms, the differential evolution algorithm.
• The mutation strategy of the algorithm is modified to work with the current solution, the global best solution, and a random solution. The resulted mutated vector from this procedure is used to produce a new modified crossover solution.
• The crossover procedure is self-adapted to cover low locality and high locality based on the iteration number, where in case of the odd iterations, the high locality is applied to obtain more diversity, and in case of the even iterations the low locality is applied to obtain local neighbor solutions. The comparison is done with the classical version of the algorithm, and the results show efficiency in terms of CPU time, evaluation, and accuracy.

## Graphical abstract

A graphical abstract is mandatory. The graphical abstract should summarize the contents of your article in a concise, pictorial form. Authors must provide images that clearly represent the work described in the article. Graphical abstracts should be submitted as a separate file. Image size: ***please provide an image with a minimum of 531 × 1328 pixels (h × w) or proportionally more***. The image should be readable at a size of 5 × 13 cm using a regular screen resolution of 96 dpi. Preferred file types: TIFF, EPS, PDF or MS Office files.

**Figure:**
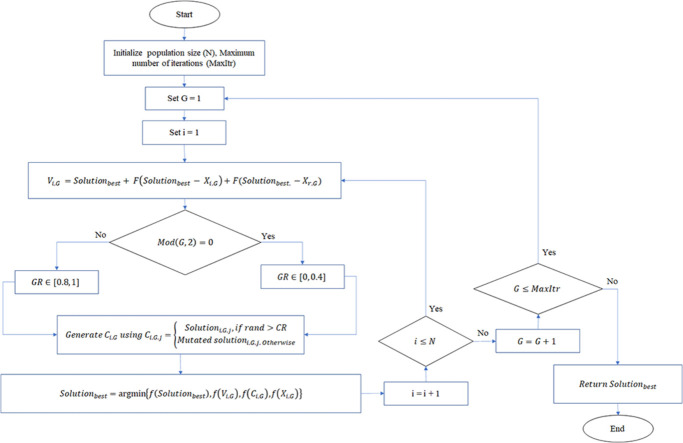

Specifications table

**Table:** 

Subject area:	Computer Science
More specific subject area:	Metaheuristics and Evolutionary Algorithms
Name of your method:	Modified Differential Evolution Algorithm for Solving Optimization Problems
Name and reference of original method:	Storn, R., & Price, K. (1997). Differential evolution–a simple and efficient heuristic for global optimization over continuous spaces. Journal of global optimization, 11(4), 341-359
Resource availability:	*https://github.com/sadeer1966/A-Modified-Differential-Evolution-Algorithm-Based-on-Improving-A-New-Mutation-Strategy-and-Self-Adap*

**Method details**

## Introduction

There are many approaches for solving optimization problems. Some of them are called classical approaches that rely on using mathematics, such as the simplex method. These classical approaches can easily deal with low-scale problems and produce exact optimal solutions. However, when the problem is NP-hard, classical approaches encounter a large number of variables and constraints, making them very difficult to apply. Therefore, heuristics and metaheuristic approaches could be ideal in such cases. The word ``metaheuristic'' consists of two ancient Greek words. The first one is ``meta,'' which means upper-level methodology, and the second one is ``heuristic,'' which means the art of discovering new strategies. Therefore, metaheuristics are upper-level methodologies that lead to obtaining approximate optimized solutions by using guiding strategies [1]. Metaheuristics in research are classified into single-based and population-based metaheuristics. In single-based metaheuristics, each iteration focuses on having a random solution in the solution space, and it searches around it for new neighbours using local search, hoping to find better local solutions. The global best solution is the best solution found throughout all iterations. In the case of population-based metaheuristics, the approach begins by generating a population of random solutions, followed by mutation procedures that change the positions of the included solutions in the population. The main concept is to cover more solution areas in the solution space by having diversified solutions in the population. In each solution area, local search is performed to find the best local solution. This paper presents a new modification to one of the population-based metaheuristics named the differential evolution algorithm (DE). The paper is organized as follows: the second section presents a literature review on DE, the third section describes the modified DE, the fourth section discusses numerical experiments that optimize the parameters of the proposed modified DE, and finally, the fifth section presents the conclusion and future research points.

## Literature Review

Qin et al.,[2] proposed a self-adaptive DE (SaDE) algorithm, in which both trial vector generation strategies and their associated control parameter values are gradually self-adapted by learning from their previous experiences in generating promising solutions. Zhang and Sanderson [3] proposed a new mutation strategy "DE/current-to pbest" with optional external archive and adaptively updating control parameters. The DE/current-to- pbest is a generalization of the classic "DE/current-to-best", while the optional archive operation utilizes historical data to provide information on progress direction. Both operations diversify the population and improve the convergence performance. Islam et al. [4] proposed a new mutation strategy with binomial crossover and two adaptive control parameters. In their work, a biased selection method is introduced where the mutant vector undergoes crossover with ‘p’ top-ranked population vectors, rather than the target vector. The authors validated the results on 25 CEC 2005 benchmark problems. Reynoso-Meza et al.,[5] used a local search routine to improve convergence and an adaptive crossover operator. Tanabe & Fukunaga [6] proposed a new parameter adaptation technique for DE by using a historical memory of successful control parameter settings in order to guide the selection of future control parameter values. This technique has been evaluated depended on the comparison of 28 problems from the CEC2013 benchmark set.

Xia & Wang [7] proposed a novel Self adaptive Differential Evolution algorithm (SaDE), where the two control parameters of F and CR in addition to the choice of learning strategy are not required to be pre-specified. Tanabe & Fukunaga [8] proposed L-SHADE that extended the SHADE with Linear Population Size Reduction (LPSR). According to the linear function, it has been continually decreased the population size. Fan and Zhang [9] proposed self-adaptive differential evolution with adaptive crossover strategies. Brest et al. [10] presented a differential evolution algorithm (iL-SHADE) which is an improved version of the well- known L-SHADE algorithm. It has been used for solving single objective real parameter optimization problems. Awad et al. [11] proposed algorithm called (LSHADE-EpSin), they used a new ensemble sinusoidal approach in order to adapt automatically the values of the scaling factor of the Differential Evolution algorithm. Brest et al. [12] presented a new algorithm, namely jSO. The algorithm is an improved variant of the iL-SHADE algorithm, mostly it was conjugated with a new weighted version of mutation strategy. Stanovov et al. [13] proposed a new variant of LSHADE algorithm. The basic idea of it is to adapt its mutation strategy by using selective pressure. The experiments were done on CEC 2018 benchmark functions. Stanovov et al. [14] proposed a new parameter control scheme for the differential evolution algorithm. Song et al.[15] presented an enhanced success history adaptive DE with greedy mutation strategy (EBLSHADE) was employed in order to optimize the parameters of PV models to propose a parameter optimization method. Shen et al. [16] proposed a modified jSO algorithm (MJSO) that had a significant impact on the performance of the algorithm. It was based on cosine similarity with parameter adaptation and a novel opposition-based learning restart mechanism.

Most of the previously developed DE algorithms are efficient in terms of convergence speed and simplicity. The previous work on DE algorithms has primarily focused on modifying mutation strategies and implementing self-adaptation. Mutation strategies contribute to achieving a greater diversity of solutions, while self-adaptation processes help reduce the number of trials required in the experimental design by minimizing the number of parameters. This paper presents a new mutation strategy that utilizes the position of the best solution found, the position of the current solution, and another randomly selected solution from the population. This strategy is named DE/current-to-best/2. In the proposed mutation strategy, the new position is calculated proportionally to the best solution found by multiplying a random weight by the distance between the current solution and the best, and another random weight by the distance between the randomly selected solution and the best. This can lead to more diverse solutions and expedite the convergence towards the best solution found. The other modification of the DE algorithm in this paper introduces a new self-adaptation strategy based on the crossover procedure of the algorithm. This crossover procedure involves generating either a new diverse solution using a higher crossover probability, which allows for more characteristics from the mutated solution found by DE/current-to-best/2 or generating a new solution that incorporates more characteristics from the current solution. Additionally, the paper presents a design of experiments in order to optimize the parameters of the newly modified algorithm, which is rarely found in other works.

## Differential Evolution Algorithm

As aforementioned, the differential evolution algorithm is one of the population-based metaheuristics. The classical version of the DE algorithm begins with an initial population that consists of random solutions, each with a corresponding vector, to cover diversified areas of the solution space. Thereafter, the algorithm has three phases: mutation, crossover, and selection. The next subsections provide information about these phases and their modifications.

## Mutation Phase

In this phase, the vectors of the population solutions are to be mutated using mutation strategies at each iterationG. To illustrate some of the strategies, the following notations are used.

Notations:

**Table:** 

$V_{i,G}$	The mutated vector
$X_{r_{j},\;G}$	Random vector j from population
$X_{i,G}$	Vector iin iteration G
$X_{best,\;G}$	Global best vector in iteration G
$C_{i,G}$	The crossover vector of vectoriin iteration G
F	Scaling factor

The most common mutation strategies are found in Deng et al. [17] as follows:

(1) DE/rand/1
  (1) $$V_{i,\;G\;}=X_{r1,\;G\;}+\;F\;\left(X_{r2,\;G\;}-\;X_{r3,G}\right)$$
(2) DE/best/1
  (2) $$V_{i,\;G\;}=X_{best,\;G\;}+\;F\;\left(X_{r1,G\;}-\;X_{r2,G}\right)$$
(3) DE/current-to best/1
  (3) $$V_{i,\;G\;}=X_{i,\;G\;}+\;F\left(X_{best,\;G\;}-\;X_{i,\;G}\right)+F\left(X_{r1,\;G\;}-\;X_{r2,G}\right)$$
(4) DE/rand/2
  (4) $$V_{i,\;G\;}=X_{r1,\;G\;}+\;F\left(X_{r2,\;G\;}-\;X_{r3,\;G}\right)+F\;\left(X_{r4,\;G\;}-\;X_{r5,G}\right)$$
(5) DE/best/2
  (5) $$V_{i,\;G\;}=X_{best,\;G\;}+\;F\left(X_{r1,\;G\;}-\;X_{r2,\;G}\right)+F\;\left(X_{r3,\;G\;}-\;X_{r4,G}\right)$$

The new proposed strategy in this paper named as DE/current-to-best/2, which is shown in Eq. (6). The strategy utilizes the distance between the position of the best vector and the current. In addition, another random vector is to be used with the best vector to extend the modification of the mutated vector. The parameter F is chosen to be a normally distributed random number with a mean of 0 and a standard deviation of 0.5. As mentioned earlier, this strategy enhances diversity by determining a new position based on the distances between the current solution and the best solution found, as well as between a randomly selected solution from the population and the best solution found. The parameter F is selected as a normally distributed random number to incorporate both negative and positive values, which contributes to increased diversity in the mutated vector's components.

(6) $$V_{i,\;G\;}=X_{best,\;G\;}+\;F\left(X_{best,\;G\;}-\;X_{i,\;G}\right)+F\left(X_{best,\;G\;}-\;X_{r,G}\right)$$

### Crossover Phase

In this phase, the algorithm generates a new solution that shares characteristics from positions $X_{i,G}\;$and $V_{i,\;G\;}$using crossover probabilityCR. Each element jin the crossover vector $C_{i,G}$ can be generated as follows:

(7) $$C_{i,G,j}=\left\{ \begin{array}{c} X_{i,G,j},if\;rand>CR\\ V_{i,G,j},Otherwise \end{array}\right.$$

The random number, rand, in Eq. (7) is uniformly distributed random number with the interval [0,1]. The crossover probability plays a role of either having diversity or locality. The higher the crossover probability the higher the diversity, and the lower the crossover probability the higher chance of having a local neighbor solution. In this paper, the proposed crossover probability is to be generated using the iteration number, where the odd iteration number allows for CRgenerated randomly from the interval[0.8,1], and for the even iteration number, CRis to be generated randomly from the interval[0,0.4]. So, the odd iterations allow for higher diversity solutions and the even iterations allow to have local neighbor solutions. In the case of odd iterations, the generated crossover rate (CR) is very high and difficult to reach due to the interval including large numbers between 0.8 and 1. Consequently, during odd iterations, most of the components of $V_{i,G}$ are selected to create the crossover vector. This leads to the creation of a crossover vector that inherits more characteristics from $V_{i,G}$, resulting in greater diversity. Conversely, during even iterations, the CR value is very low, indicating that most of the components of the crossover vector are selected from $X_{i,G}$, resulting in a new vector that can be described as a local neighbor of $X_{i,G}$. In summary, odd iterations promote diverse solutions, while even iterations produce local neighbors for the current solution.

### Selection Phase

In the selection phase, the best solution found among $X_{i,G}$, $V_{i,G}$, and $C_{i,G}$ will be selected to remain in the population of the next iteration. In case that the evaluations of all of them are the same, then the vector that will remain the population in the next iteration is to be randomly selected from the three vectors.

**Fig. 1:**
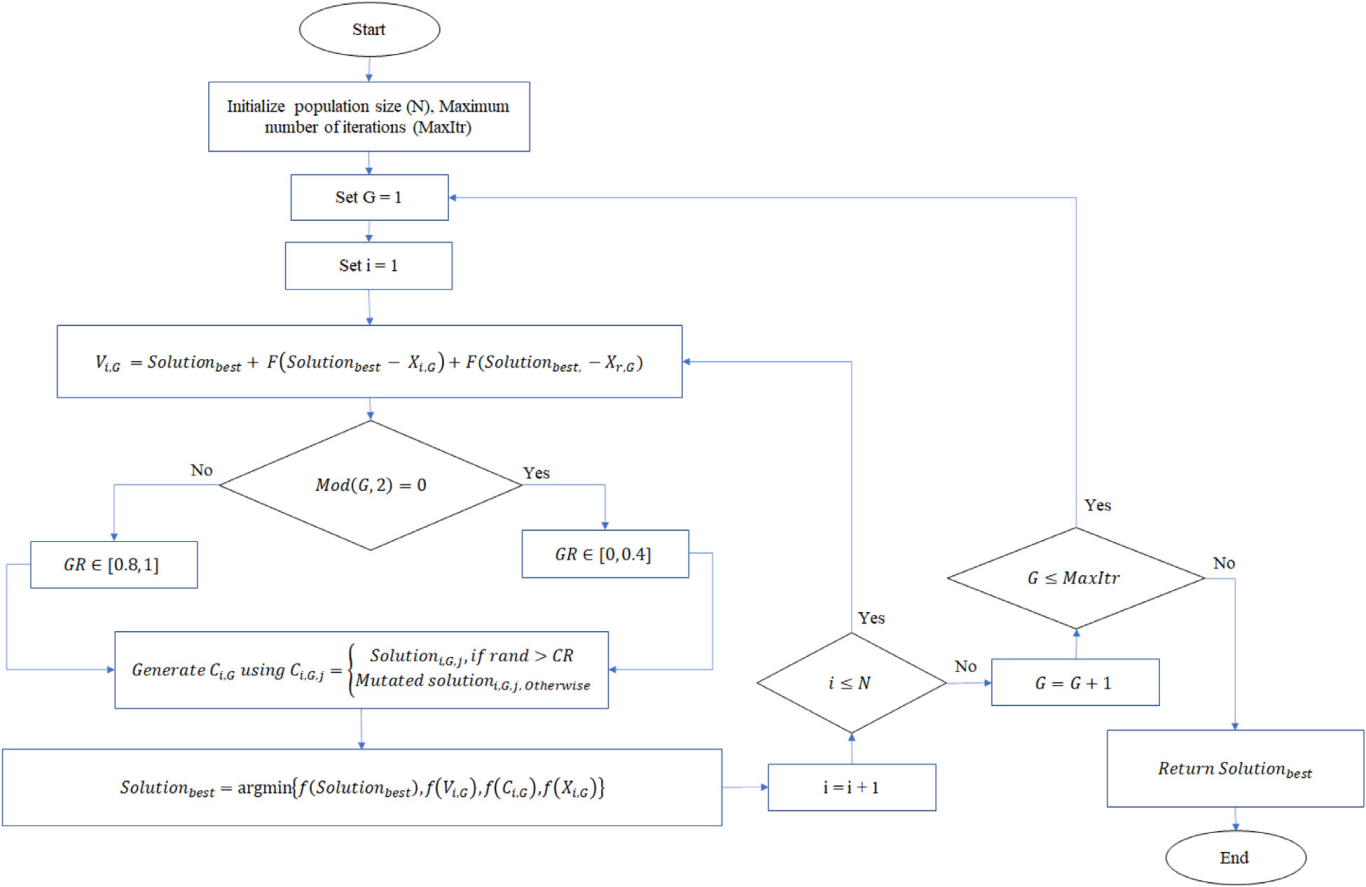
The flowchart of the proposed DE algorithm

Now all the phases of the proposed modification of the DE algorithm are discussed. Fig. 1 shows the flowchart the new proposed DE algorithm. The source code of the algorithm can be found in https://github.com/sadeer1966/A-Modified-Differential-Evolution-Algorithm-Based-on-Improving-A-New-Mutation-Strategy-and-Self-Adap.

To further illustrate the steps of the modified algorithm, the following pseudo code is provided. Additionally, both the modified DE and classical DE algorithms are coded in Python and can be accessed a thttps://github.com/sadeer1966/A-Modified-Differential-Evolution-Algorithm-Based-on-Improving-A-New-Mutation-Strategy-and-Self-Adap.

The pseudo code of the Proposed algorithm is as follows:

**Table:** 

1	$Generate\;initial\;population\;with\;N\;random\;solutions\;and\;store\;the\;best\;in\;Solution_{best}$
2	SetG=1
3	While G≤MaxItr do:
4	Seti=1
5	Whilei≤Ndo:
6	$V_{i,G\;}=X_{best,G}+F\left(X_{best,G\;}-X_{i,G}\right)+F\left(X_{best,G\;}-X_{r,G}\right)$
7	Ifmod(G,2)=0do:
8	GenerateCR∈[0,0.4]
9	Else:
10	GenerateCR∈[0.8,1]
11	$enerate\;C_{i,G}\;u\sin g\;C_{i,G,j}=\{\begin{array}{c} X_{i,G,j},\inf rand>CR\\ V_{i,G,j},Otherwise \end{array}$
12	$Solution_{best}=argmin\left\{f\left(Solution_{best}\right),\;f\left(V_{i,G}\right),f\left(C_{i,G}\right),f\left(X_{i,G\;}\right)\right\}$
13	i=i+1
14	G=G+1
15	$Return\;Solution_{best}$

## Numerical Experiments

In this section, 11 optimization problems have been selected to test the modified DE algorithm. The evaluation is based on the output of the functions and the CPU time ($CPU_{time}$). The problems are listed in Table 5. This paper proposes two parameters that can be calculated throughout the implementation of the algorithm, which are the scaling factor F that is generated using uniformly distributed random number and the self-adapted crossover probability CR. The remaining parameters for the proposed DE algorithm are the population size (P) and the number of iterations (N). The population size and number of iterations in any metaheuristic play a crucial role in convergence. Therefore, these two parameters are to be optimized using design of experiments. The selected levels for both the Pand Nparameters are 10, 30, 50, 70, and 90 solutions and iterations, respectively. The full factorial design consists of multiple factors. Each factor has a set of discrete levels. The experiments in the full factorial design are done according to listing all combinations of these levels across their factors [18]. Therefore, the full factorial design herein can be represented as in Table 1.

**Table 1:** Full Factorial Design

Trails	Population	Iterations
1	10	10
2	10	30
3	10	50
4	10	70
5	10	90
6	30	10
7	30	30
8	30	50
9	30	70
10	30	90
11	50	10
12	50	30
13	50	50
14	50	70
15	50	90
16	70	10
17	70	30
18	70	50
19	70	70
20	70	90
21	90	10
22	90	30
23	90	50
24	90	70
25	90	90

The experimental design in this paper aims to enhance the efficiency of the algorithm by optimizing the evaluation of the test optimization functions and the CPU time required by the proposed DE algorithm. Therefore, the response value ($R_{i}$) for any trail i, which is required to be minimized, is calculated according to Eq. (8), where f(x)is the evaluation of the test optimization function.

(8) $$R_{i}=\;f\left(x\right)-\frac{1}{1+CPU_{time}}$$

**Fig. 2:**
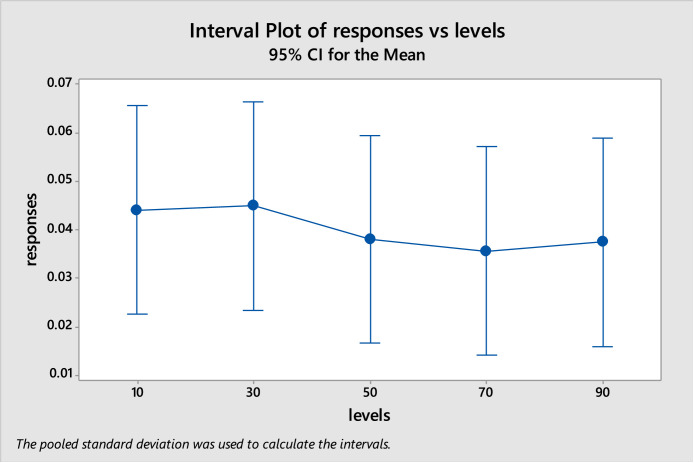
Interval plot for population levels

**Fig. 3:**
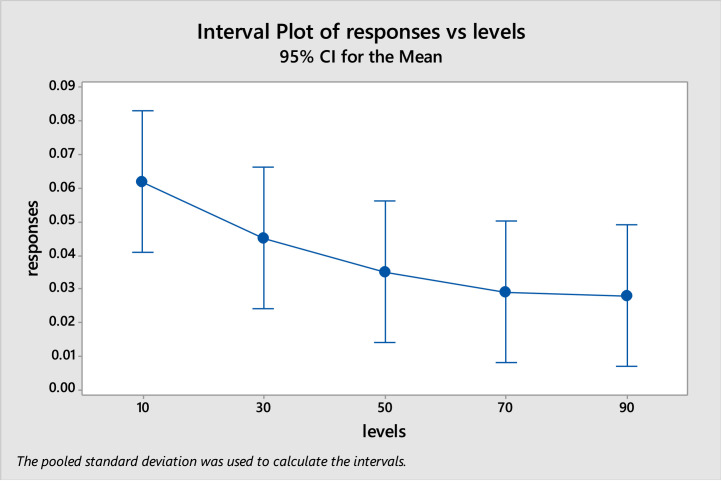
Interval plot for iteration levels

After running the 25 trails contained in Table 1, the response value for each trail i is normalized using Eq. (9) for each test optimization function and these normalized values ($N_{i}$) are found as in Table 6 [19]. The analysis of variance (ANOVA) is used to find if there are any differences between levels of the population and iteration factors. After implementing ANOVA using Minitab, it found that the P-value of the population levels equals to 0.964, which means there is no significant difference between the population levels. The P-value of the iteration levels equals to 0.146, which also means that there is no significant difference between the iteration levels. In case of the population levels, the interval plot of responses vs levels as in Fig. 2 shows that all levels are the same without any further investigation. But in case of the iteration levels, the interval plot of responses vs levels as in Fig. 3 shows that some levels may be investigated between the level 10 and 30. So, the suggestion herein is to keep the population size equals to 10 and make another experiment that include only the iteration factor with levels 10, 15, 20, 25, and 30. After implementing the experiment and applying ANOVA, it found that the P-value of iteration factor equals to 0.029, which means that not all of its levels come from the same population and there is a significant difference between some or all of these levels. Therefore, Tukey's pairwise comparison test is done to find which levels differ.

(9) $$N_{i}=\;\frac{R_{i}}{\sum_{i=1}^{25}R_{i}}$$

**Fig. 4:**
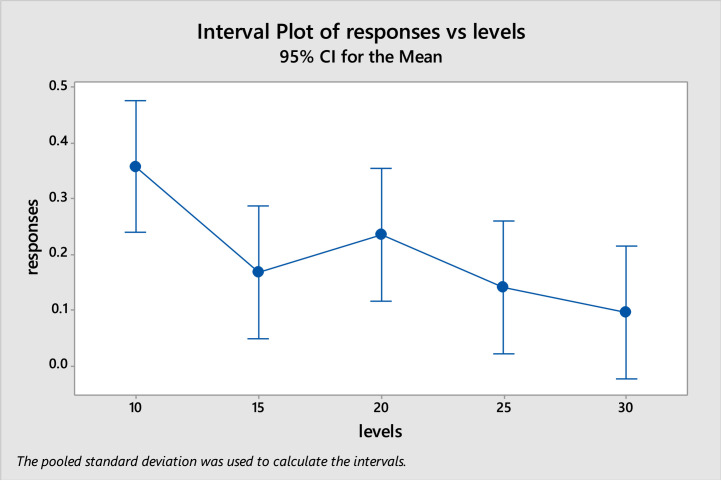
Interval plot of iterations after applying Tukey test

So, after applying the Tukey's pairwise comparison test, the grouping information shows two groups that differ from one another, which are A and B. Table 2 shows the grouping information and the interval plot is shown as in Fig. 4. Since the grouping information shows that level 10 differs from level 30 and level 30 shows better response than level 10, the selected level for iteration factor is 30 iterations.

**Table 2:** Grouping information

levels	N	Mean	Grouping
10	11	0.3581	A
20	11	0.2362	A B
15	11	0.1674	A B
25	11	0.1418	A B

In summary, according to the experimental design, it can be concluded that the optimized parameters for population can be selected from any level, and in case of the number of iterations factor, the selected parameter level is 30 iterations. The comparative results section shows the results of implementing the proposed DE algorithm with its optimized parameters on 11 test optimization functions. The comparison is done with three different versions of the classical DE algorithm.

## Comparative Results

**Fig. 5:**
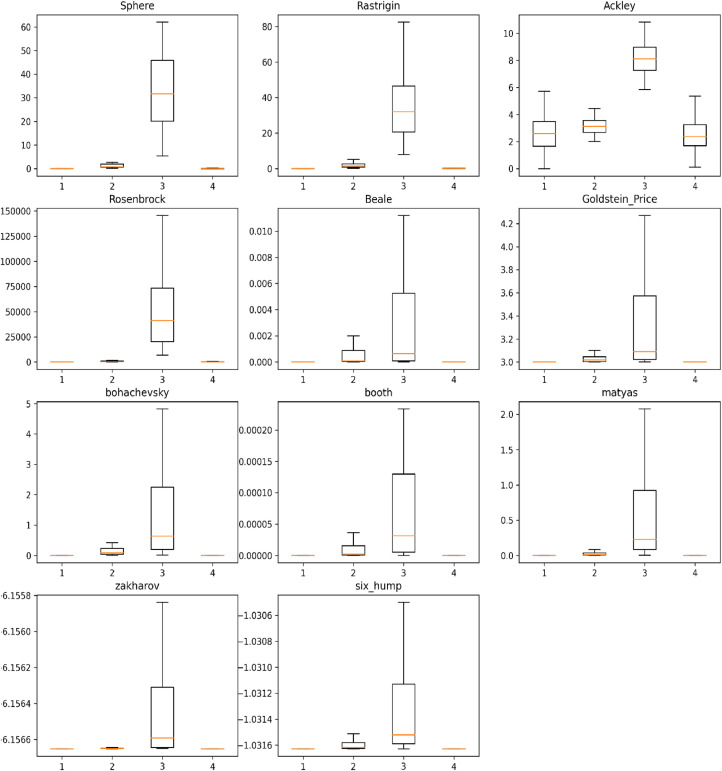
Comparative results box plots.

In this section, the comparative results are done between three different types of the classical version of DE and modified DE. All of them are coded using python programming. The three different versions used in this comparison are based on the mutation strategies in Eqs. (3), (4), and (5). Because the classical version of DE doesn't have adaptation of CR, The CRvalue considered in this comparison equals 0.25. To obtain comparative results, 50 outputs were obtained from each algorithm using the 11 optimization functions listed in Table 5 . Fig. 5 depicts the box plots of the output from each algorithm. It is evident that the modified DE yields similar results to the classical DE, which employs a mutation strategy based on Eq. (5). Both of these methods outperform the remaining algorithms. The box plots also indicate that both the modified DE and the classical DE exhibit lower variability compared to the other algorithms. Table 3 displays the mean comparative results of the output from each algorithm, revealing that the modified DE algorithm outperforms the others in terms of mean results, where the bold text highlights the best outputs. In order to assess the robustness of the results, the standard deviations of the outputs were calculated and are presented in Table 4. The standard deviation values of the modified DE algorithm demonstrate its superior robustness compared to the other algorithms.

**Table 3:** The mean comparative results

Functions	Modified DE	Classical 1	Classical 2	Classical 3
Sphere	**0**	1.349530703	33.90031884	0.20725885
Rastrigin	**6.11304E-05**	2.039027697	35.26733858	0.19439251
Ackley	2.850293399	3.23459056	8.028763444	**2.636296377**
Rosenbrock	**23.8655269**	1061.1744	61128.32002	234.258577
Beale	0.10212286	**0.002883156**	0.011259602	0.11556722
Goldstein_Price	4.62	**3.111058471**	4.721087403	8.40000001
bohachevsky	**0**	**0**	6.50017E-06	**0**
booth	**0**	1.62335E-05	0.000150294	**0**
matyas	**0**	**0**	1.54294E-05	**0**
zakharov	**-6.15665237**	-6.15664587	-6.156279425	-6.15665237
six_hump	**-1.03162845**	-1.03156355	-1.031129072	-0.99898186

**Table 4:** The standard deviation comparative results

Functions	Modified DE	Classical 1	Classical 2	Classical 3
Sphere	**0.000238**	1.496218	17.319566	0.787995
Rastrigin	**0.000156**	1.971238	17.990258	0.839166
Ackley	1.543311	**0.979291**	1.362058	1.560347
Rosenbrock	**34.063151**	2312.820948	59452.559248	310.251986
Beale	0.192298	**0.008944**	0.032390	0.214863
Goldstein_Price	11.340000	**0.432722**	4.946090	17.076299
bohachevsky	**0**	3.68222E-07	0.000023	2.78769E-14
booth	**0**	0.000044	0.000301	7.24439E-11
matyas	**1.15774E-31**	0.000001	0.000058	1.27292E-11
zakharov	**8.88178E-16**	0.000014	0.000688	8.33903E-11
six_hump	**0**	0.000152	0.000878	0.159935

**Table 5:** Test Optimization Functions

Function name	Function	Global minimum	Limits
Sphere	$f\left(x\right)=\;\sum_{i=1}^{d}x_{i}^{2}$	$f\left(x^{*}\right)=0,\;at\;x^{*}=\left(0,\;0,\;\ldots ,\;0\right)$	$x_{i}\in \left[-5.12,\;5.12\right]$
Rastrigin	$f\left(x\right)=\;10d+\sum_{i=1}^{d}\left[x_{i}^{2}-10\;\text{cos}\left(2\pi x_{i}\right)\right]$	$f\left(x^{*}\right)=0,\;at\;x^{*}=\left(0,\;0,\;\ldots ,\;0\right)$	$x_{i}\in \left[-5.12,\;5.12\right]$
Ackley	$f\left(x\right)=\;-20\;e^{-0.2\;\sqrt{0.50\;(x^{2}+y^{2})}}-e^{0.5(\cos2\pi x+\cos2\pi y)}+e$	$f\left(x^{*}\right)=0,\;at\;x^{*}=\left(0,\;0,\;\ldots ,\;0\right)$	$x_{i}\in \left[-32.768,\;32.768\right]$
Rosenbrock	$f\left(x\right)=\;\sum_{i=1}^{d-1}\left[100+{\left(x_{i+1}-\;x_{i}^{2}\right)}^{2}+{\left(x_{i}-1\right)}^{2}\right]$	$f\left(x^{*}\right)=0,\;at\;x^{*}=\left(1,\;1,\;\ldots ,\;1\right)$	$x_{i}\in \left[-5,\;10\right]$ $x_{i}\in \left[-2.048,\;2.048\right]$
Beale	$f\left(x\right)={\left(1.5-x_{1}+x_{1}x_{\left(2\right)}\right)}^{2}+{\left(2.25-x_{1}+x_{1}x_{2}^{2}\right)}^{2}+{\left(2.625-x_{1}+x_{1}x_{2}^{3}\right)}^{2}$	$f\left(x^{*}\right)=0,\;at\;x^{*}=\left(3,0.5\right)$	$x_{1},x_{2}\in \left[-4.5,\;4.5\right]$
Goldstein-Price	$\begin{array}{c} f\left(x\right)=\left[1+{\left(x_{1}+x_{2}+1\right)}^{2}\left(19-14x_{1}+3x_{1}^{2}-14x_{2}+6x_{1}x_{2}+3x_{2}^{2}\right)\right]\\ {}*[30+{\left(2x_{1}-3x_{2}\right)}^{2}\left(18-32x_{1}+12x_{1}^{2}+48x_{2}-36x_{1}x_{2}+27x_{2}^{2}\right)] \end{array}$	$f\left(x^{*}\right)=3,\;at\;x^{*}=\left(0,\;-1\right)$	$x_{1,}x_{2}\in \left[-2,\;2\right]$
Bohachevsky	$f_{1}\left(x\right)=\;(x_{1}^{2}+2x_{2}^{2}-0.3\cos\left(3\pi x_{1}\right)-0.4\cos\left(4\pi x_{2}\right)+0.7$ $f_{2}\left(x\right)=\;x_{1}^{2}+2x_{2}^{2}-0.3\cos\left(3\pi x_{1}\right)\cos\left(4\pi x_{2}\right)+0.3$ $f_{3}\left(x\right)=\;x_{1}^{2}+2x_{2}^{2}-0.3\cos\left(3\pi x_{1}+\;4\pi x_{2}\right)+0.3$	$f\left(x^{*}\right)=0,\;at\;x^{*}=\left(0,\;0\right),for\;all\;j=1,2,3$	$x_{i}\in \left[-100,\;100\right]\forall i=1,2$
Booth	$f\left(x\right)=\;{\left(x_{1}+2x_{2\;}-7\right)}^{2}+{\left(2\;x_{1}+\;x_{2}-5\right)}^{2}$	$f\left(x^{*}\right)=0,\;at\;x^{*}=\left(1,\;3\right)$	$x_{1,}x_{2}\in \left[-10,\;10\right]$
Matyas	$f\left(x\right)=\;0.26{\left(x_{1}^{2}+x_{2}^{2}\right)}^{2}-0.48x_{1}x_{2}$	$f\left(x^{*}\right)=0,\;at\;x^{*}=\left(0,\;0\right)$	$x_{1,}x_{2}\in \left[-10,\;10\right]$
Zakharov	$\sum_{i=1}^{d}x_{i}^{2}+{\left(\sum_{i=1}^{d}0.5ix_{i}\right)}^{2}+{\left(\sum_{i=1}^{d}0.5ix_{i}\right)}^{4}$	$f\left(x^{*}\right)=0,\;at\;x^{*}=\left(0,\;0,\;\ldots ,\;0\right)$	$x_{i}\;\in \;\left[-5,\;10\right],\;\forall i=1,\ldots ,d$
Six hump camel	$f\left(x\right)=\left(4-2.1x_{1}^{2}+\frac{x_{1}^{4}}{3}\right)x_{1}^{2}+x_{1}x_{2}+\left(-4+4x_{2}^{2}\right)x_{2}^{2}$	$f\left(x^{*}\right)=-1.0316,\;at\;x^{*}=\left(0.0898,\;-0.7126\right)and\;\left(-0.0898,0.7126\right)$	$x_{1}\in \left[-3,\;3\right]$ $x_{2}\in \left[-2,\;2\right]$

**Table 6:** Normalized results

Sphare	Rastrigin	Ackley	Rosenbrock	Beale	Goldstein_Price	Bohachevsky	Booth	Matyas	Zakharov	Six_Hump
0.072	0.502	0.047	0.065	0.029	0.273	0.044	-0.865	0.045	0.037	-0.022
0.055	0.051	0.047	0.026	0.043	0.382	0.028	0.075	0.045	0.041	0.047
-0.008	0.017	0.046	0.001	0.049	0.198	0.051	0.091	0.045	0.041	0.047
-0.022	-0.007	0.045	0.000	0.049	0.003	0.050	0.101	0.044	0.041	0.046
0.025	0.022	0.045	0.011	0.047	0.005	0.049	0.099	0.044	0.041	0.046
0.175	0.077	0.047	0.463	0.038	0.030	0.032	-0.029	0.045	0.041	0.036
0.043	0.004	0.044	0.006	0.039	0.002	0.047	0.097	0.044	0.041	0.045
-0.007	0.056	0.042	0.001	0.046	0.001	0.046	0.095	0.042	0.041	0.045
-0.004	0.001	0.041	0.031	0.045	0.001	0.044	0.092	0.041	0.040	0.044
-0.014	-0.007	0.039	0.004	0.042	0.001	0.042	0.089	0.039	0.040	0.043
0.166	0.088	0.046	0.162	0.029	0.024	0.050	-0.003	0.045	0.041	0.036
0.071	0.033	0.042	0.027	0.046	0.003	0.046	0.088	0.042	0.041	0.045
-0.017	-0.003	0.039	0.006	0.043	0.001	0.042	0.090	0.039	0.040	0.043
-0.009	-0.001	0.036	0.000	0.041	0.003	0.040	0.084	0.037	0.040	0.043
-0.017	-0.006	0.034	0.002	0.038	0.001	0.038	0.080	0.035	0.040	0.042
0.135	0.061	0.045	0.168	0.037	0.048	0.010	0.098	0.044	0.040	0.045
0.010	0.006	0.040	0.002	0.042	0.006	0.044	0.091	0.040	0.040	0.044
-0.014	0.002	0.036	0.001	0.040	0.002	0.040	0.084	0.037	0.040	0.042
-0.009	0.005	0.033	0.001	0.037	0.002	0.036	0.077	0.034	0.040	0.041
-0.015	-0.004	0.031	0.001	0.034	0.001	0.033	0.067	0.032	0.039	0.040
0.341	0.106	0.044	0.015	0.044	0.004	0.048	0.099	0.043	0.040	0.041
0.048	0.008	0.039	0.005	0.040	0.005	0.042	0.087	0.039	0.040	0.043
0.011	0.000	0.034	0.002	0.037	0.001	0.037	0.079	0.035	0.040	0.041
-0.010	-0.003	0.031	0.001	0.035	0.002	0.032	0.071	0.032	0.039	0.040
-0.005	-0.005	0.028	0.002	0.032	0.001	0.030	0.065	0.029	0.039	0.038

## Conclusion

This paper presents a new modification of the DE algorithm in both the mutation and crossover phases. The mutation procedure generates new positions using the distance between the position of the current solution and the position of the best solution found. Additionally, it utilizes the distance between the position of the best solution and a randomly selected position from the population. This type of mutation procedure allows for the generation of neighboring solutions between three positions and converges towards the best solution found. The crossover procedure controls the locality around the position of the current solution based on the iteration number. During odd iterations, the locality becomes higher, resulting in greater diversity. Conversely, during even iterations, the locality decreases, generating local neighbors around the position of the current solution. The scaling factor and the crossover probability are self-adapted in the proposed modified DE. To optimize the parameters of the proposed DE, the design of experiments is conducted with only two factors: the number of iterations and the population size. After optimizing these parameters, comparative results are obtained for 11 optimization problems, comparing the results of the classical version of DE with the modified version. The comparative results demonstrate the high efficiency of the modified DE. It is expected that the modified version of DE will be utilized in future research to address the following problems:

- Job shop scheduling problem
- Warehouse location problem
- Aircraft landing problem
- Timetable scheduling problem

## CRediT author statement

Sadeer Fadhil: Conceptualization, Methodology, Software, Validity tests, Data curation, Writing- Original draft preparation, Visualization, Investigation. Hegazy Zaher, Naglaa Ragaa, and Eman Oun: Supervision

## Declaration of Competing Interest

The authors declare that they have no known competing financial interests or personal relationships that could have appeared to influence the work reported in this paper

## Data Availability

The data is already included inside the manuscript. I addition, the code developed is uploaded to GitHub with referring to its link inside the manuscript.
